# Drivers of post-adoption of e-wallet among academics in Palestine: An extension of the expectation confirmation model

**DOI:** 10.3389/fpsyg.2022.984931

**Published:** 2022-09-21

**Authors:** Ahmad Daragmeh, Adil Saleem, Judit Bárczi, Judit Sági

**Affiliations:** ^1^Doctoral School of Economics and Regional Studies, Hungarian University of Agriculture and Life Sciences, Gödöllő, Hungary; ^2^Faculty of Finance and Accountancy, Budapest Business School, Budapest, Hungary

**Keywords:** continuous intention, e-wallet adoption, expectation confirmation model (ECM), security, trust

## Abstract

E-wallet is one of the latest innovations in the field of payments. However, despite numerous studies on the adoption of e-finance systems, the post-adoption phase is largely neglected. In this paper, we use the extended Expectation Confirmation Model (ECM) to address this gap by focusing on the study of consumers’ continuous intentions regarding the use of an e-wallet service. We conducted an electronic questionnaire-based survey among 503 e-wallet users in Palestine. Using structural equation modeling to analyze the conceptual model of the study, our results confirm that satisfaction, trust, and perceived usefulness have a significant impact on consumers’ continuous intention regarding e-wallet. In addition, the study found that perceived security has an insignificant impact on consumer satisfaction. The study has several implications: E-wallet providers should improve their services in terms of performance, privacy, and security to ensure customer loyalty in this competitive industry.

## Introduction

Rapid advances in the information and communications industry and the proliferation of smart devices have spurred the digitization of the financial sector and the development of various electronic payment options, including electronic wallets, as an alternative to traditional physical money (Bezhovski, 2016; Daqar et al., 2021). In Palestine, the Monetary Authority gives high priority to financial technology. It promotes the shift to electronic payments, believing that electronic payment services play an essential role in accelerating financial inclusion and providing financial services to unbanked people (PMA, 2020). Cash is still the most common method in the financial activities of Palestinian consumers. However, the Palestinian Monetary Authority (PMT) expects that licenses for electronic payment services to banks and fintech companies will rapidly boost e-commerce. It is also likely that the high internet penetration rate among Palestinians, which stood at 70.6% in January 2021, could play a crucial role in achieving this goal (KEMP, 2021).

E-wallet is sometimes referred to as m-wallet or digital wallet. For simplicity, the term e-wallet is used hereafter to refer to all types of digital wallets. e-Wallet is a service that allows financial activities such as money transfers or purchases to be made through a specific electronic device, such as a smartphone or computer (Singh et al., 2017). The use of e-wallets appears to benefit all players in the e-wallets ecosystem, such as consumers, banks, FinTech companies, and digital service providers. E-wallets provide consumers with the ability to check their account balances and transactions, make payments at any time, conduct secure transactions within a short period of time, and receive offers and discounts (Yang et al., 2021). For their part, banks and FinTech companies benefit from transaction costs, brand image, and improved value-added services for their customers.

Meanwhile, research on the use of information systems has proliferated over the past decade. Various theories such as the Theory of Planned Behavior (TPB) (Ajzen, 1991), Technology Acceptance Model (TAM) (Davis, 1989), and Innovation Diffusion Theory (IDT) (Rogers, 1995) have been drawn upon to understand the factors that influence consumers’ intention to adopt information systems. In the same context, (Bhattacherjee, 2001) proposed the expectation-confirmation model (ECM) as an extension of TAM to understand users’ intention to continue using a particular system.

E-payment is one of the most widespread and transparent payment methods in developed countries and has recently been gaining popularity in developing countries such as Palestine. As the e-payment market continues to grow, it is necessary to investigate the factors that influence Palestinian consumers’ intention to continue using the e-wallet service. Most of the studies conducted so far on the financial technology sector in Palestine focused on the factors that influence the adoption and actual use of e-banking services (Maitah and Hodrab, 2015; Ayyash, 2017; Salem et al., 2019; Sulaiman and AbdelKarim, 2019). It was found that there is a deficit in capturing the factors that influence consumers’ intention to continue using e-wallet services, which are considered as a new service for the financial technology industry in the country. To accurately understand consumer behavior and intentions regarding continued use of e-wallet services in the future, this study incorporates two factors into the ECM: Trust and Perceived Security. The purpose of this study is to address the gaps and shortcomings of studies on the continued use of e-wallets and to examine the relationship between trust and perceived security and the intention to continue using e-wallet services in Palestine. Therefore, this study examines the factors that influence the continued use of e-wallets using the extended ECM. After the introduction, Section 2 explains the theoretical background of our study, and Section 3 presents the conceptual model. The methodology of the study is described in Section 4. The results of the study are presented in Section 5. Sections 6 and 7 address the discussion and implications of the study. The final section addresses limitations and future research.

## Theoretical background

### Status of digital financial payments (e-Wallet) in Palestine

Palestine is a modest economy, with low- to middle-income status and a population of 5.1 million. The service sector is the engine of the Palestinian economy, accounting for 60 percent of gross domestic product (GDP) and a significant portion of employment in 2019. In fact, more than one-third of the population is employed in the service sector and 23% in trade (*PCBS, 2021*). Meanwhile, the financial sector is an essential component of the Palestinian economic system, influencing and being influenced by its development and transformation (Daragmeh and Barczi, 2021).

Through basic transaction accounts, digital payment services can promote greater adoption of digital financial services. Access to and use of digital payment systems are critical enablers of the digital economy. Low-income and rural households, as well as women and youth who are often underserved by traditional financial services, can gain greater access to digital financial services. The digital financial sector in Palestine fits into the broader framework of the Middle East and North Africa (MENA) region, which has experienced phenomenal expansion in recent years (Daqar, 2021). The fact that the total number of mobile connections in Palestine exceeds 86% in 2021 (up from 64% in 2011) and the penetration rate of internet users has reached 71% bodes well for the future of the digital finance sector in general and e-wallet users in particular (*Mobile for Development, 2021*).

Although several banks and FinTech companies offer mobile payment services, Internet banking, and SMS banking that can facilitate the provision of various digital financial services to customers, the adoption rate of these services remains low. Providers of electronic wallets in Palestine are banks and FinTech companies, and they offer common services, including: Payment for purchases at various stores, the ability to pay fees and subscriptions at (municipalities, offices, and insurance companies), cash withdrawals from the digital wallet *via* ATMs, money transfer services between customers’ digital wallets, withdrawal and deposit services from the digital wallet account (cash deposits and withdrawals), and checking the balance of the digital wallet account. Meanwhile, the use of e-wallets continues to increase, with person-to-person payments and cash withdrawals accounting for the majority of transactions, and a smaller proportion of payments for services and e-shopping. The total amount held on prepaid cards appears to be proportionally larger than that held on electronic wallets. In the Palestinian market, many banks, telecom providers, and FinTech companies offer e-wallet services. Google Pay and Palpay are the most popular payment methods among Palestinians (Ibrahim et al., 2021).

The PMT has laid the groundwork for the development of digital financial services and has recently issued regulations and guidelines to encourage the growth of this sector. For example, the National Strategy for Financial Inclusion (2018–2025) includes regulations to support the use of e-money and initially focuses on prepaid cards and e-wallets. In addition, regulations for the licensing of payment service providers (2018) have been issued, allowing non-banks to access the Palestinian market and increasing competition in the sector. In addition, PMT has adopted a comprehensive national strategy for the development of payments. The strategy covers the years 2018 to 2023 and has the overall goal of making Palestine a leading user of electronic payment systems to promote the national economy and improve risk mitigation. The strategy promotes the adoption of electronic payments through the participation of all key stakeholders, with a particular focus on the related infrastructure. The selected plan focuses primarily on promoting digital payments; it will help lay the groundwork for broader adoption of digital financial services in Palestine. The strategy also focuses on developing the legal environment to support payments and e-commerce; developing the infrastructure to support payments; improving access to e-payments, especially for diverse populations; raising public awareness of e-payments; and using the public sector in Palestine as a catalyst to increase the use of e-payments (World Bank Group, 2021).

### Expectations confirmation model

Oliver, R. I. developed expectations confirmation theory (ECT) in 1980 as a fundamental theory for measuring consumer satisfaction with the purchase/use of a particular product or service (Oliver, 1980). According to expectation disconfirmation theory, customer satisfaction or dissatisfaction results from a discrepancy between the customer’s expectations and perceived performance, and the confirmation of these expectations is a good predictor of overall satisfaction (Tzeng et al., 2021). The SERVQUAL model (Parasuraman et al., 1985) links the confirmation of expectations to the quality level of the service as the discrepancy between the customer’s expectation of the service provided and the customer’s perception of the service received. When quality expectation exceeds quality perception, it leads to higher perception, i.e., satisfaction with service quality is higher than expected (Tzeng et al., 2021). ECT is often used in a marketing context to assess consumer satisfaction and post-purchase behavior. In ECT, consumers’ repurchase decision begins when they have an initial expectation of the product or service. After the initial consumption, they express an opinion about the performance of the product/service and compare it with the initial expectations (Halilovic and Cicic, 2013). The level of satisfaction is achieved when the expectation matches the perceived performance. Thus, satisfied customers have the intention to buy the product again, while dissatisfied customers are discouraged from doing so. In other words: After using the product or service for a while, the consumer gains experience and a better understanding of the product or service’s performance, leading to new cognition. Confirmation occurs when the consumer compares this cognition to their previous expectation to determine if the evaluation is identical. If the actual performance of the service/product is higher than the consumer’s expectation, it is a positive confirmation. If the actual performance is lower than the expectation, it is a negative confirmation (Oliver, 1980).

Bhattacherjee has developed ECM by integrating the TAM and the ECT to study the continuous user behavior related to information systems (Bhattacherjee, 2001). The degree of expectation confirmation can influence the user’s sense of value and satisfaction with an information system, thus increasing the intention to continue using the system. In other words, the higher the degree of expectancy confirmation, the greater the perceived usefulness of the system, the greater the user’s perceived satisfaction, and the greater the user’s intention to continue using the information system. Although user expectations are a broad concept, user expectations at TAM include perceived usefulness and perceived ease of use. However, (Bhattacherjee, 2001) believes that perceived usefulness is a sufficient expectation for continued use of an information system, as it is the only belief that has been shown to influence user intent at any stage of an information system use. The ECM emphasizes post-adoption expectations over pre-adoption expectations. The more expectations users have about a particular information system, the more experience they gain. After processing these experiences, a user’s expectations for using that information system may change (Edwards et al., 2019). (Bhattacherjee, 2001). suggests that post-adoption expectations significantly affect a user’s satisfaction with a particular information system. Moreover, perceived usefulness is a surrogate measure of post-use expectancy and is consistent with the ECT definition of expectancy as an individual belief (Davis, 1989). He also claimed that the ECM does not account for the performance variable because it assumes that the confirmation and satisfaction variables mediate the effect of the performance. Finally, he argued that the ECM explains how the causal relationship between (expectation disconfirmation and dissatisfaction) leads to acceptance discontinuity of information systems (Bhattacherjee, 2001).

Several researchers have used ECM to investigate users’ intentions to continue using various information systems services. Lee incorporated TAM in ECM into an empirical model to interpret and predict users’ intention for continuous e-learning (Lee, 2010). Shang used an extension of ECM by incorporating perceived value into the model to examine the factors that influence consumers’ intention to continuously use mobile shopping apps (Shang and Wu, 2017). Susanto developed an extended ECM framework that incorporated self-efficacy, perceived security and privacy, and trust to predict Korean users’ intention to continuously use smartphone banking services (Susanto et al., 2016). Nevertheless, C.C developed an integrated model between ECM and Health Belief Model (HBM) to test continuity intention related to mobile payments during the pandemic COVID-19 (Sreelakshmi and Prathap, 2020). This work can validate the model in a new technological context by extending ECM to e-wallet services. This approach is consistent with the philosophy of knowledge construction. Therefore, ECM can provide a solid foundation for the development of our research model.

### Trust and perceived security

Trust refers to the development of a positive perception based on reliability and dependence on a person or system (Rotter, 1980). The concept of trust extends beyond the interpersonal realm to include interaction with technology as a critical factor influencing user behavior related to technology adoption (Kuriyan and Ray, 2009; Smith, 2010). Several studies (Safari, 2012; Sevim and Eroğlu Hall, 2014; Bauman and Bachmann, 2017) focusing on the marketing of online services have found that trust is an essential factor for online customers. Some even believe that the absence of trust means that e-commerce is doomed to extinction or, at best, stagnation (Harris and Goode, 2010). This is because trust is an important factor that a consumer considers when contemplating a financial transaction over the Internet. Trust is so important because e- wallet users have to share their sensitive personal and financial information with e- wallet providers (Chawla and Joshi, 2019). Therefore, users may be very concerned about security and privacy when using such applications. Therefore, trust mechanisms are important to shift risks and support users’ decision to continue using online financial services rather than those that require physical contact (Chawla and Joshi, 2019). Previous research has highlighted the critical role of security and privacy in the context of online financial services (Casaló et al., 2007; Mohamrned et al., 2016; El Haddad et al., 2018; Leong et al., 2020). The influence of security is not limited to the initial stage of technology use and acceptance, but also extends to the user’s intention to continue using a particular technological system (Kumar et al., 2018). Susanto found that a lack of security and privacy reduces user satisfaction and Trust in smartphone banking (Susanto et al., 2016). In this study, trust and security were integrated into ECM because security is the basis for users’ trust in an e- wallet. Moreover, trust plays an important role in the user’s intention to continue using the e- wallet.

### Research model and hypotheses

Building on the theoretical foundation of ECM for measuring continuous use of information systems, we developed an ECM for studying continuous intent to use e- wallets. We extended the original ECM framework to include perceived trust and perceived security. We consider continuous intention to use e- wallets as an indication of the user’s intention to use e- wallets in the future. Satisfaction is the perceived overall feeling of using a product or service (Chow and Shi, 2014). In relation to IS, satisfaction is expected to increase users’ intention to continue using the system (Limayem et al., 2007). Therefore, higher perceived satisfaction could lead to using IS again in the future. According to ECT theory, user satisfaction positively affects their intention to continue using the same IS (Brown et al., 2014). This relationship has been confirmed in several studies (Susanto et al., 2016; Khayer and Bao, 2019; Chiu et al., 2020; Pang et al., 2020; Rahi et al., 2020; Sreelakshmi and Prathap, 2020). Bhattacherjee argued that user satisfaction is critical to post-adoption behavior, including intentions for continued use (Bhattacherjee, 2001). At the same time, Chuah discussed that consumer satisfaction has a critical impact on consumer loyalty to a particular product or service (Chuah et al., 2017). In this study, we hypothesize that perceived satisfaction has a positive influence on consumers’ intention to continue using e-Wallet. Therefore, we hypothesize the following:

**H1.** Perceived satisfaction has a positive effect on the intention to continue using e-Wallet.

Perceived usefulness is the extent to which a person believes that using a particular IS would improve their job performance (Daragmeh et al., 2021a). By using IS, users can benefit in many ways, such as increased accuracy, efficiency, and speed in completing tasks (Yang et al., 2009). A study by Rahi confirmed that perceived usefulness has a significant positive impact on users’ intention to continue using internet banking (Rahi et al., 2020). Foroughi also confirmed the significant positive influence of perceived usefulness on m-Banking users’ satisfaction (Foroughi et al., 2019). Several other studies have demonstrated the positive relationship between perceived usefulness and intention to continue using and between perceived usefulness and perceived user satisfaction (Susanto et al., 2016; Khayer and Bao, 2019; Chiu et al., 2020; Shiau et al., 2020; Sreelakshmi and Prathap, 2020). Based on previous literature, we hypothesize that the more benefits users derive from the e-wallet, the more satisfied they are with them and the more likely they are to continue using them. Accordingly, the following hypotheses were developed:

**H2.** Perceived usefulness has a positive effect on the intention to continue using e-Wallet.

**H3.** Perceived usefulness has a positive effect on user satisfaction.

Consumers are more likely to form a strong bond with a service provider if they trust it. The stronger this bond is, the more impossible it is for consumers to sever this relationship (Ponder et al., 2016). Trust is the desire to establish a bond with a particular service based on positive expectations about the future behavior of those service providers (Zhou, 2013). Trust in using an electronic wallet reflects the user’s belief in the reliability of that service. Thus, when users develop trust in an electronic wallet, they may transfer their trust to their continued use of this type of service. Trust gives users confidence that they will receive a safe and high-quality service in the future because they trust that service providers have the ability and competence to provide them with high-quality services (Zhou, 2013). Previous studies have shown that trust is an important determinant of a user’s intention to continue using a particular information system (Gao et al., 2015; Hsu et al., 2015).

In addition, trust also has a positive impact on user satisfaction (Susanto et al., 2016). The more users express their trust in the e-wallet service, the more satisfied they are with the service. Therefore, we hypothesized the following:

**H4.** Trust has a positive effect on the intention to continue using e-Wallet.

**H5.** Trust has a positive effect on user satisfaction.

The uncertainty associated with new payment technologies with high participation leads to privacy and data security concerns. Perceived security (PS) refers to the extent to which a user believes the Internet is safe when using a particular online payment channel (Chawla and Joshi, 2019). Security plays an essential role in maintaining the relationship between the merchant, the user, and the payment system in this digital world (Kar, 2020). In e-commerce, security is generally considered a threatening factor that could cause certain conditions, events, or circumstances that may lead to economic difficulties in the form of data loss, information destruction, misuse, fraud, and possibly alteration of original data (Salimon et al., 2015). Literature has shown that users’ satisfaction and Trust in a digital system decreases when they have concerns about security and privacy in such a system (Ofori et al., 2017; Kumar et al., 2018). Kumar found that perceived security significantly impacts Indian consumers’ Trust in using m-Wallets (Kumar et al., 2018). However, since e-Wallets involve the storage and transfer of personal and financial information, they raise more security concerns than traditional payment methods. Therefore, we hypothesize that the greater the users’ perception of security, the greater their Trust and satisfaction in using e-Wallets in the future.

**H6.** Perceived security has a positive effect on perceived satisfaction.

**H7.** Perceived security has a positive effect on perceived Trust.

Confirmation is insofar as the current usage experience confirms the original expectations (Oghuma et al., 2016). ECM indicates that confirmation of expectations positively influences perceived usefulness and satisfaction with IT products and services (Bhattacherjee, 2001). This is consistently confirmed by previous studies (Khayer and Bao, 2019; Chiu et al., 2020; Pang et al., 2020; Rahi et al., 2020; Shiau et al., 2020). According to Bhattacherjee (2001), the higher the level of expectation confirmation, the more useful the system is to the user, the more satisfied the user is with the system, and the higher the user’s intention to continue using such a system. The uncertainty associated with new, highly engaged payment technologies raises privacy and data security concerns (Slade et al., 2015; Khalilzadeh et al., 2017). Digital payment providers, therefore, need to put security mechanisms in place to protect customers from third-party attacks (Hoffmann and Birnbrich, 2012; Khalilzadeh et al., 2017). The perceived threat is minimized once the actual usage experience validates the security measures, so the overall perceived security is improved after adoption (Bhattacherjee and Barfar, 2011; Apanasevic et al., 2016).

Moreover, a study by Susanto found that post-usage confirmation has an important impact on users’ perceptions of using mobile banking services, including perceived usefulness and perceived security (Susanto et al., 2016). It also has a positive influence on user satisfaction. Based on the preceding discussion, we hypothesize the following:

**H8.** Confirmation has a positive effect on perceived satisfaction.

**H9.** Confirmation has a positive effect on perceived usefulness.

**H10.** Confirmation has a positive effect on perceived security.

**FIGURE 1:**
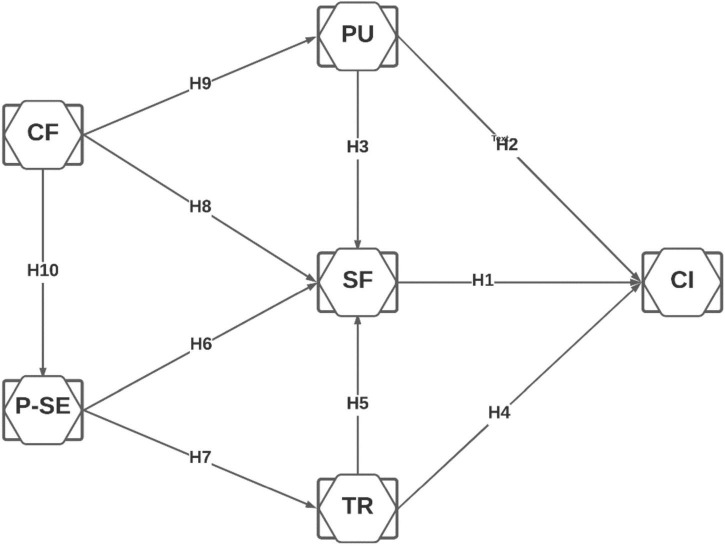
Research model. CF, confirmation; PU, perceived usefulness; SF, satisfaction; P-SE, perceived security; TR, trust; CI, continuous intention.

Based on the hypotheses developed above, Figure 1 shows our research model.

## Methodology

### Measurement of constructs

To test the hypotheses, a two-part survey was conducted. The first part consists of a series of measurable items in the research framework to measure the theoretical constructs, and the second part captures the demographic data of the sample. The items in the survey were taken from various previously published sources to ensure content validity. Perceived usefulness was measured with items adapted from a study by Daragmeh et al. (2021b). The items measuring confirmation, satisfaction, and users’ continuous intention were adopted from Daragmeh et al. (2021b). The indicators measuring trust were adopted from Kumar et al. (2018). Finally, the items measuring perceived security were adopted from the study of Gao et al. (2015). A five-point Likert scale ranging from “strongly disagree” to “strongly agree” was used to measure the items. Table 2 shows the measurement items.

### Data collection analysis

The study targeted e-Wallet users in Palestine. Data were collected from students, faculty, and administrators at Al-Quds Open University, a Palestinian public university. It comprises one-third of Palestinian university students and has 19 branches in different parts of the country. The university applies the (Blending Learning Model), which is a blend of open learning and e-learning. To ensure representativeness, the proportion of respondents was set according to the proportion of eligible groups in the population. Thus, the university environment represents different subcultures and values (Chandler et al., 2021). Nevertheless, it is at the forefront of e-service provision (Kim-Soon et al., 2014). Therefore, it can be assumed that this segment is inclined to use e-services in their daily lives.

A total of 740 responses were received, of which 503 were used for further analysis. 137 responses were excluded because respondents indicated that they had no experience with e-Wallet transactions. (58.5%) of the respondents were male, and 41.5% were female. The majority of the respondents were under 25 years of age (60.6%), followed by respondents between 25 and 39 years of age (27.9%). Most respondents were undergraduate students (82.3%) with a bachelor’s degree (64%). In addition, the majority of respondents (87.1%) had more than 1 month of experience with e-Wallet services. A detailed description of the demographic characteristics of the respondents can be found in Table 1. The hypothesis was tested using partial least squares (PLS) with SmartPLS 3.3 software. Due to its predictive function, PLS was preferred in this study to evaluate how well exogenous constructs predict endogenous constructs (Hair et al., 2019a). This study followed a two-step analytic approach, first examining the measurement model and then the structural model (Foroughi et al., 2019).

**TABLE 1:** Respondents demographics.

	N	%
**Sex**		
Male	294	58.5
Female	209	41.5
Total	503	100%
**Age**		
Less than 25	305	60.6
25 to 40	140	27.9
40 to 55	41	8.1
Above 55	17	3.3
Total	503	100%
Education level		
Bachelor	322	64
Master	116	23.1
PhD	19	3.8
Others	46	9.1
Total	503	100%
**Occupation**		
Student	414	82.3
Lecturer	27	5.4
Administrator	39	7.7
Others	23	4.6
Total	503	100%
E-Wallet usage experience		
Less than one a month	65	12.9
1 to 6 months	193	38.4
More than 6 months	245	48.7
Total	503	100%

### Common method bias

We applied the Harman factor test to investigate whether a possible common method bias (CMB) was present in our study. We performed a principal axis factor (PAF) analysis to determine the critical number of factors describing variance (Mvududu and Sink, 2013). The results show that the total variance was well below the suggested 50%, and the single construct explained 34.85% of the variance (Podsakoff et al., 2003). We also assessed the CMBs using the VIF values of the constructs that resulted from the full collinearity tests. The results are also shown in Table 3. The values were also lower than the proposed 3.3 (Kock, 2015). Thus, the CMB was not perceived as a threat in this study.

## Results

### Measurement model

Exploratory analysis can be assessed by examining the scale reliability, convergent validity, and discriminant validity of the model to test validity and reliability (Hair et al., 2019a). To test reliability, we assessed indicator loadings, composite reliability, average variances extracted, and Cronbach’s alpha. The loading range of the indicators was between 0.768 and 0.883, as shown in Table 2. Thus, all the loadings were above 0.70, indicating the reliability of the indicators. It can also be seen that all constructs met the criteria of convergent validity (Chin, 1998). The composite reliability values ranged from 0.842 to 0.893, which is above the threshold of 0.70 (Fornell and Larcker, 1981). The AVE and Cronbach α-values were also above the threshold of 0.7 (Hair et al., 2019b). To assess discriminant validity, we examined the intersection of the measured items and the square root of AVE. Table 3 shows that the correlation between items does not exceed the square root of AVE. From the analyses, we conclude that we have a reliable and valid measurement model.

**TABLE 2:** Results of the measurement model analysis.

	Loading	(α)	(CR)	(AVE)
Confirmation (CF)		0.821	0.893	0.736
CF1: My experience of using e-Wallet service exceeded my expectation.	0.883			
CF2: The service level offered by the e-Wallet provider exceeded my expectation.	0.845			
CF3: Overall, most of my expectations from using e-Wallet were confirmed.	0.845			
Perceived Security (P-SE)		0.721	0.842	0.640
P-SE1: I am concerned over the security of personal information exchange on e-Wallet.	0.771			
P-SE2: use of e-Wallet is safe and secure	0.785			
P-SE3: e-Wallet payments maintain privacy	0.843			
Perceived Usefulness (PU)		0.730	0.848	0.650
PU1: using e-Wallet would enable me to pay more quickly	0.810			
PU2: using e-Wallet make it easier for me to conduct payments.	0.768			
PU3: I would find e-Wallet a useful possibility for paying.	0.839			
Trust (TR)		0.769	0.866	0.683
TR1: The e-Wallet service provider is trustworthy	0.791			
TR2: The e-Wallet service providers keep their promise	0.821			
TR3: The e-Wallet service providers keep customers’ best interests in mind	0.866			
Satisfaction (SF)		0.750	0.857	0.667
SF1: I feel satisfied with e-Wallet usage.	0.850			
SF2: I feel contented with e-Wallet usage.	0.776			
SF3: I feel happy using the e-Wallet service.	0.822			
Continuous Intention (CI)		0.759	0.862	0.675
CI1: I intend to continue using e-Wallet rather than discontinue its use.	0.820			
CI2: I intend to continue using e-Wallet than using any alternative means.	0.830			
CI3: if I could, I would like to continue using e-Wallet as much as possible.	0.815			

**TABLE 3:** Discriminant validity- average variance extracted (AVE) values.

	CF	CI	P-SE	PU	SF	TR
CF	0.858					
CI	0.579	0.822				
P-SE	0.294	0.361	0.800			
PU	0.471	0.708	0.344	0.806		
SF	0.713	0.696	0.283	0.579	0.817	
TR	0.416	0.595	0.568	0.557	0.501	0.827

### Structural model

**FIGURE 2:**
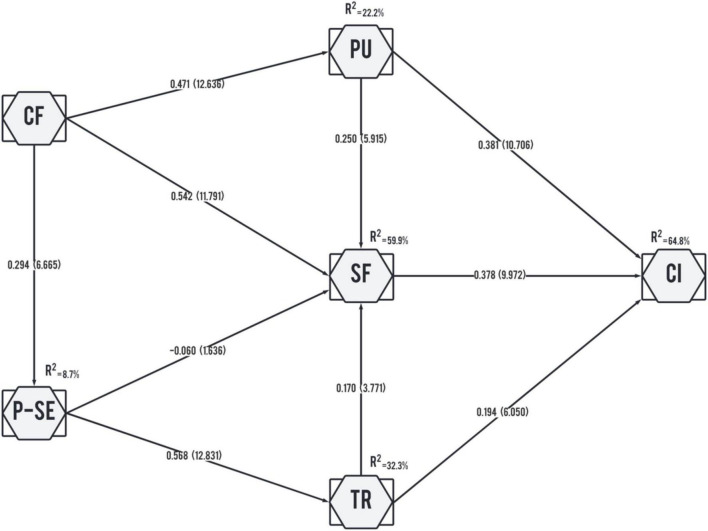
Hypotheses test results.

Before evaluating the structural model, the presence of multicollinearity must first be established using the VIF. Table 3 shows no collinearity problem as the collinearity between constructs is below a threshold of 5.0 (Hair et al., 2017). The structural model was examined using a bootstrapping procedure with 5,000 subsamples to test the hypotheses. The significance and correlation of each hypothesized pathway and the explained variance are important for the structural model analysis. As shown in Figure 2, the model explained 64.8% of the variance for continuous intention to use e- wallets, 59.9% of the variance for satisfaction, 32.2% of the variance for trust, 22.2% of the variance for perceived usefulness, and 0.087% of the variance for perceived security. The results of the path coefficient and the relationship between the constructs are shown in Table 4. Confirmation has a significant positive effect on satisfaction (β 0.542, p 0.000), perceived usefulness (β 0.471, p 0.000), and perceived security (β 0.294 p 0.000). Perceived security has a significant positive effect on trust (β 0.568, p 0.000). However, the results show that the effect of perceived security on satisfaction is insignificant (β−0.060, p 0.102). Perceived usefulness had significant positive effects on satisfaction (β 0.250, p 0.000) and continuous intention (β 5 0.381, p 0.000). Trust also had similar effects on satisfaction (β 0.250, p 0.000) and continuous intention (β 0.194, p 0.000). Finally, the results show the significant positive effect of satisfaction on users’ continuous intention toward e-wallets (β 0.378, p 0.000). Therefore, all hypotheses except H6 are supported.

**TABLE 4:** Hypotheses testing.

No.	Hypothesis	Original sample (O)	Sample mean (M)	Standard deviation (STDEV)	T-Statistics (|O/STDEV|)	*P*-values	Status
H1	SF - > CI	0.378	0.377	0.038	9.972	0.000	Accepted
H2	PU - > CI	0.381	0.380	0.036	10.706	0.000	Accepted
H3	PU - > SF	0.250	0.248	0.042	5.915	0.000	Accepted
H4	TR - > CI	0.194	0.196	0.032	6.050	0.000	Accepted
H5	TR - > SF	0.170	0.171	0.045	3.771	0.000	Accepted
H6	P-SE - > SF	−0.060	−0.059	0.036	1.636	0.102	Rejected
H7	P-SE - > TR	0.568	0.570	0.044	12.831	0.000	Accepted
H8	CF - > SF	0.542	0.542	0.046	11.791	0.000	Accepted
H9	CF - > PU	0.471	0.473	0.037	12.636	0.000	Accepted
H10	CF - > P-SE	0.294	0.296	0.044	6.665	0.000	Accepted

Furthermore, we evaluated effect sizes (f^2^) to examine the strength of the relationship between the constructs. Cohen suggested that the *p*-value can show that the effect exists but does not reveal the effect size (Cohen, 1988). The f^2^ values of (0.02, 0.15, and 0.35) are considered small, medium, and substantial effect sizes. The results of the effect size analysis presented in Table 5 show that the effect sizes of all exogenous variables with their endogenous variables except the effect of (perceived security on satisfaction) were correspondingly positive and above 0.02. Predictive relevance (Q^2^) was also assessed using the blindfolding procedure. The blindfolding procedure should only be used for endogenous constructs with a reflective measurement, as confirmed by Hair et al. The acceptable value of Q^2^ should be above 0, indication that the model is predictive (Cohen, 1988; Hair et al., 2017). Table 5 shows that the predictive relevance of continuous intention and satisfaction is substantial, and that of perceived trust is medium. In contrast, the predictive relevance of perceived usefulness and perceived security is low.

**TABLE 5:** Computing effect size analysis f^2^ and predictive relevance Q^2^.

Construct	R^2^	Q^2^	f^2^	Decision
CI	0.648	0.432		
SF			0.250	Small
TR			0.069	Small
PU			0.235	Medium
SF	0.599	0.394		
TR			0.038	Small
PU			0.096	Small
P-SE			0.006	Small
CF			0.453	Substantial
TR	0.323	0.215		
P-SE			0.476	Substantial
PU	0.222	0.142		
CF			0.286	Medium
P-SE	0.087	0.057		
CF			0.095	Small

## Discussion

The research objective described in the introduction was to investigate the effects of trust and perceived security and confirmation, perceived usefulness, and satisfaction on the continuity of e-Wallet use in Palestine. As shown in Table 5, all hypotheses except H6 are supported. The results also show that perceived security has a significant impact on trust but not on user satisfaction. The literature has previously confirmed the irrefutable role of perceived security in building trust (Kim et al., 2010; Salimon et al., 2015; Ejdys et al., 2019). Because the use of e- wallets involves sensitive financial data, it is important to reassure users that financial transactions *via* digital devices are secure (Leong et al., 2020). Only when users have a high level of Trust in the security of an e-wallet provider will they use its e-Wallet services with Trust. The insignificant impact of perceived security on user satisfaction contradicts our expectations and is inconsistent with previous studies (Gupta et al., 2020). Since security is considered a must in financial services, this could be a reason for its low influence on user satisfaction. However, their absence may also have a negative impact on user satisfaction. e-Wallets allow users to conduct financial transactions without interacting with them. Therefore, users may perceive a higher level of risk and uncertainty compared to offline financial services. Therefore, user satisfaction is important before they decide to continue using e-wallet services (Jiaxin Zhang et al., 2019).

Similar to previous studies (Bhattacherjee and Barfar, 2011; Shang and Wu, 2017), our study found that confirmation significantly impacts perceived security when using e-Wallets. This suggests that confirmation of usage experiences consistent with pre-use expectations implies improved perceived security of the e-Wallet after use, which mitigates risk perceptions toward the service (Oghuma et al., 2016). Consistent with other studies (Zhou, 2013; Kumar et al., 2018; Talwar et al., 2020), perceived usefulness, perceived trust, and perceived satisfaction emerged as the most important determinants of e-Wallet usage continuity in our study. Perceived usefulness had the greatest influence on intention to use an e-Wallet (Foroughi et al., 2019). It also had a significant impact on user satisfaction, supporting previous work (Susanto et al., 2016; Foroughi et al., 2019; Rahi et al., 2020). It can be argued that it is important that users find e-Wallet applications highly useful so that once they use them, they develop a sense of satisfaction with all their features, which also affects the positive intention to continue using this service (Bhattacherjee, 2001; Foroughi et al., 2019; Rahi et al., 2020; Sreelakshmi and Prathap, 2020). The significant positive impact of satisfaction on intention to continue use can be explained by users’ willingness to continue using e-Wallets only when they are satisfied with the features.

Moreover, our results showed that the path coefficient of satisfaction with user confirmation is larger than that of satisfaction with perceived usefulness. These results differ from the results of other previous researches (Foroughi et al., 2019; Ruangkanjanases et al., 2020). Meanwhile, Nascimento et al. confirmed no significant difference in the effect of confirmation and perceived usefulness on smartwatch users’ satisfaction (Nascimento et al., 2018). We, therefore, argue that more attention should be paid to the differences between users of financial systems such as electronic wallets and other non-financial systems. For example, e-Wallet users pay more attention to confirming their expectations than the perceived performance after use to influence emotional reactions and intention to continue using the e-Wallet (Bhattacherjee, 2001).

Several studies on financial systems have confirmed the significant positive effect of confirmation on perceived usefulness (Foroughi et al., 2019; Rahi et al., 2020). This means that in the post-adoption phase, positive confirmation tends to increase the belief in the usefulness of e-Wallets. Based on this, e-Wallet service providers should focus on improving users’ experiences with positive confirmation of their initial expectations of using e-wallets to increase their satisfaction and perception of the functional benefits. To ensure confirmation and improve user satisfaction, providers must deliver on their promises before offering such services. Managers need to devote more resources to improving their R&D initiatives to ensure that e-wallet systems include all the features and functions desired by users. In this context, managers could consider involving users in further developing services and applications to improve confirmation and perceived usefulness, increasing user satisfaction.

Trust, the other factor in ECM, had a significant positive impact on customer satisfaction and intention to continue using e-Wallets. The results are consistent with previous studies on electronic financial systems (Bricci et al., 2016; Koloseni and Mandari, 2017; Ofori et al., 2017; Kumar et al., 2018; Poromatikul et al., 2019). When e-wallet users develop confidence that transactions conducted in the system are secure and do not cause financial losses, this has a positive effect on satisfaction and motivates them to continue using e-wallet. However, the path coefficient of trust on continuous intention was found to be the lowest compared to perceived usefulness and satisfaction. The reason could be that trust only affects the use of e-services in the short term but not in long-term relationships (Susanto et al., 2016).

## Research contributions

### Theoretical contribution

Our study makes a theoretical and practical contribution to the literature. Theoretically, this study extends the framework provided by the ECM for understanding users’ continuous intentions regarding the use of e-wallet systems by adding two important factors (trust and perceived security) to the model. In the post-adoption phase of e-wallets, user satisfaction is key to determining ongoing intentions to use e-wallets. Thus, when trust and perceived security are added to the key determinants of satisfaction (and perceived usefulness), about 60% of the variance in user satisfaction can be interpreted. This study confirms that trust is an important predictive factor of customer behavior in the post-adoption phase of financial systems (Talwar et al., 2020). Because electronic wallets handle sensitive financial and personal information, consumers must trust such a service before adopting it. Therefore, users’ trust in electronic financial services is influenced by the security and privacy concerns that arise when using smart app-based services.

Interestingly, the effect of perceived security on trust was significant, but the effect on satisfaction was not. It is therefore very likely that perceived security predicts factors that influence behavioral intention in the short term, such as trust, and less so for factors that influence it in the long term, such as satisfaction. This could be because customers perceive the use of e-wallets as secure in the short term, but fear that their financial and personal information could be hacked in the long term. Finally, the study results could be useful for researchers who want to investigate the factors that influence the continued use of e-finance systems so that they can refer to the results of this study in their future studies.

### Managerial contribution

In practice, the more satisfied customers are, the more likely they are to continue using a particular service. Therefore, e-wallet service providers are advised to improve their services in terms of cost, performance, privacy, and security to ensure customer retention in this competitive industry. They also recommend running promotional campaigns with incentives, offers, and discounts to retain existing customers and attract potential new customers. Because perceived usefulness has the greatest influence on users’ intent to continue using them, e-wallet service providers should develop highly efficient and easy-to-use applications and launch promotional campaigns to educate current and potential customers about the importance of e-wallets and their use, as well as develop policies and programs aimed at increasing the public’s financial and technological awareness. If perceived security is a prerequisite for e-wallet users’ trust, e-wallet providers have a responsibility to develop security mechanisms that protect users’ data and money from hacking. The responsibility also extends to the government to enforce laws and regulations to ensure that e-wallet providers adhere to security and privacy criteria that protect customers’ rights. The study will help e-finance providers in Palestine understand the factors that drive consumers to continue using these services and help them develop strategies and campaigns based on this. Since the data was collected in a college setting, our findings can be shared with them. This will allow them to learn more about e-wallets and the factors that drive Palestinians to use these types of financial systems. Finally, PMT should focus on improving the regulatory environment, strengthening the national payment infrastructure, and incentivizing the use of digital financial services, and consider implementing regulatory measures to increase competitiveness. Incentivize merchants to accept e-wallets. Prioritize vulnerable areas and include new payment options in the implementation of the financial inclusion strategy. The government can also use e-wallets for subsidies and social benefits for citizens instead of making payments by check.

## Limitations and future researches

The generalizability of the results of our study is subject to certain limitations. First, there are numerous antecedents in the literature for the continued use of FinTech systems, including e-wallets. For example, perceived ease of use is a determinant of continued use of the Alipay system (Khayer and Bao, 2019), and others claim that attitude increases the explanatory power for understanding the factors that influence users’ intentions toward e-wallets (Daragmeh et al., 2021b). In addition to the hedonic value derived from the use of mobile wallets (Xavier and Zakkariya, 2021). Second, the results should be generalized with caution to other countries with different cultures. Therefore, applying the study in other countries could help identify the significant differences in intention to continue using e-wallet systems. Thus, future research will also allow us to capture the impact of cultural differences. The study was conducted in an academic setting and the questionnaire was limited to students, faculty and staff of some Palestinian universities. Therefore, the results cannot reflect the behavior of all e-wallet users in Palestine. Finally, this study used the cross-sectional method. Future research could investigate the underlying factors in a longitudinal context to get a clear picture of e-wallet users’ intention to continue using the service.

## Data availability statement

The original contributions presented in this study are included in the article/supplementary material, further inquiries can be directed to the corresponding author/s.

## Author contributions

AD and JS: conceptualization and methodology. AD: software, visualization, formal analysis, data curation, and writing—original draft preparation. JS: validation, writing—review and editing, and supervision. AS: investigation and resources. JB: project administration and funding acquisition. All authors have read and agreed to the published version of the manuscript.
